# *Aedes aegypti* in Southern Brazil: Spatiotemporal Distribution Dynamics and Association with Climate and Environmental Factors

**DOI:** 10.3390/tropicalmed8020077

**Published:** 2023-01-20

**Authors:** Joice Guilherme de Oliveira, Sérgio Antônio Netto, Edenilson Osinski Francisco, Caroline Pereira Vieira, Paula Fassicolo Variza, Betine Pinto Moehlecke Iser, Tamara Nunes Lima-Camara, Camila Lorenz, Josiane Somariva Prophiro

**Affiliations:** 1 Graduate Program in Health Sciences, University of Southern Santa Catarina–UNISUL, Avenida José Acácio Moreira, 787, Tubarão 88704-900, Santa Catarina, Brazil; 2 Graduate Program in Environmental Sciences, University of Southern Santa Catarina–UNISUL, Avenida José Acácio Moreira, 787, Tubarão 88704-900, Santa Catarina, Brazil; 3 Department of Biological and Health Sciences, University of Southern Santa Catarina–UNISUL, Avenida José Acácio Moreira, 787, Tubarão 8870d-900, Santa Catarina, Brazil; 4 Department of Epidemiology, School of Public Health, University of São Paulo, São Paulo 01246-904, São Paulo, Brazil

**Keywords:** arboviruses, climate, epidemiological control, house indices, mosquito, temperature

## Abstract

In Brazil, the mosquito *Aedes* (*Stegomyia*) *aegypti* is considered the main vector of the dengue, chikungunya, and Zika arbovirus transmission. Recent epidemiological studies in southern Brazil have shown an increase in the incidence of dengue, raising concerns over epidemiological control, monitoring, and surveys. Therefore, this study aimed at performing a historical spatiotemporal analysis of the *Ae. aegypti* house indices (HI) in southern Brazil over the last 19 years. As vector infestation was associated with climatic and environmental variables, HI data from the Brazilian Ministry of Health, climate data from the Giovanni web-based application, and environmental data from the Mapbiomas project were used in this study. Our results showed an expressive increase in the number of HI surveys in the municipalities confirming the vector presence, as compared to those in 2017. Environmental variables, such as urban infrastructure, precipitation, temperature, and humidity, were positively correlated with the *Ae. aegypti* HI. This was the first study to analyze *Ae. aegypti* HI surveys in municipalities of southern Brazil, and our findings could help in developing and planning disease control strategies to improve public health.

## 1. Introduction

In Brazil, the mosquito *Aedes* (*Stegomyia*) *aegypti* (Linnaeus, 1762) is considered the main vector involved in the transmissions of dengue (DENV), chikungunya (CHIKV), and Zika (ZIKV) arboviruses [1]. They are the etiological agents mainly responsible for the emergence and reemergence of diseases that have geographically expanded at a global scale, causing epidemics in many urban populations [2,3,4,5]. The global distribution of the *Ae. aegypti* vector is primarily restricted to tropical and subtropical areas [2]. However, factors such as climate change, urban mobility, land use, and land cover can contribute to the expansion of this vector to areas that have not registered its presence, putting billions of people at risk of DENV and other arbovirus infections [6,7,8,9]. Other factors, such as urban growth, inadequate housing conditions, deforestation, and the high adaptive potential of this species are also related to *Ae. aegypti* dispersion and, consequently, with the increased transmission of pathogens [10,11]. Urbanization has caused several changes, ranging from the loss of native vegetation in and around cities, floods, and water, soil, and air pollution to the formation of heat islands and the disappearance of important habitats of several species, including vector insects. These changes may, in turn, intensify the spread of infectious diseases [12]. In recent years, the increase in the number of dengue, Zika and chikungunya cases as well as related comorbidities have had a marked impact on public health spending due to the high mortality rate associated with these infections [13,14,15,16,17,18]. These are some of the global challenges associated with the control of *Ae. aegypti* and associated arboviruses.

In Brazilian entomological surveillance programs, health agents conduct home visits to inspect possible intra- and peri-domicile *Ae. aegypti* breeding sites, eliminate these containers, and monitor larval density data [4,19]. The Rapid Survey of *Ae. aegypti* indices (Levantamento Rapido de Indice para *Aedes aegypti*; LIRAa) uses the house (HI) and Breteau (BI) indices to assess the risk of DENV transmission. HI refers to the percentage of houses infested with *Ae. aegypti*, whereas BI refers to the number of positive containers per 100 inspected houses. Although recent studies have reported a lack of correlation between LIRAa and the incidence of arbovirus infections [20,21], LIRAa is the only official measure used by the Brazilian Ministry of Health (Ministério da Saúde; MS) to measure infestation on a regular basis in most municipalities. LIRAa has been used as an instrument for evaluating the results of control measures, rendering it possible to redirect and/or intensify some interventions or even to change previously adopted control strategies [4].

A study conducted before 2020 demonstrated that southern Brazil had the lowest incidence of dengue cases in the country [22]. However, notwithstanding the ongoing coronavirus disease 2019 (COVID-19) pandemic, Brazil recorded more than 979,000 cases of dengue (with an incidence rate of 466.2 cases per 100,000 inhabitants) in 2020. During this period, southern Brazil had the second highest incidence, with the central-western region recording the highest incidence rate [23]. Until epidemiological week 22 of 2022, 110,472 cases of dengue were recorded in Brazil, reflecting a 197.9% increase in the number of cases compared to that observed in the same period in 2021 [24,25]. The southern region had the second highest number of probable cases of dengue, with 941.7 cases per 100,000 inhabitants [25]. Three southern states showed an increase in the number of dengue, chikungunya, and Zika cases and *Ae. aegypti* infestations in 2022, in addition to the highest number of recorded deaths attributed to dengue [25]. These data highlight the need for specific analyses of the distribution of the *Ae. aegypti* vector and its relationship with climatic and environmental variables in this region. Such information may help to identify the main high-risk areas for targeting and optimizing control and surveillance actions of this vector.

The objective of this study was to analyze the distribution of *Ae. aegypti* in southern Brazil from 2003 to 2021 and to determine possible relationships between this vector and climatic and environmental variables from 2017 to 2021. The study period was determined based on the available data from the HI, the most commonly used method to analyze vector infestation in Brazilian municipalities, of *Ae. aegypti and* dengue cases.

## 2. Materials and Methods

### 2.1. Study Areas

**Figure 1:**
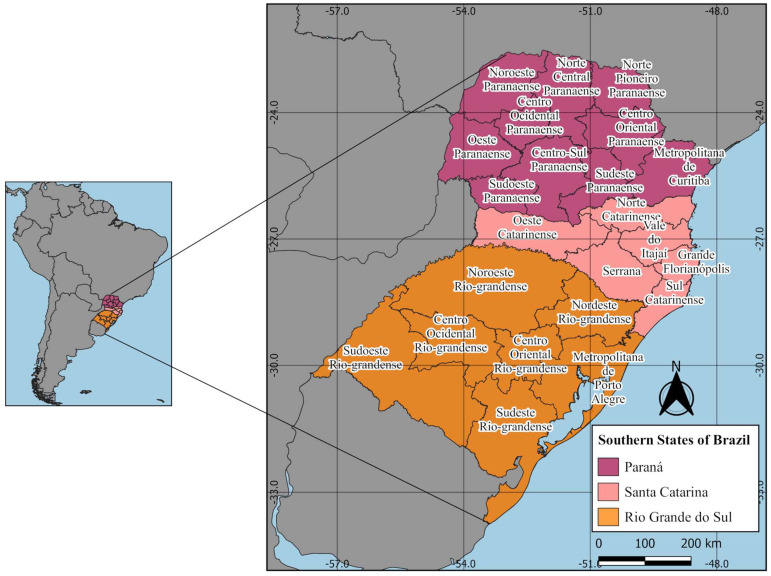
Mesoregions of southern Brazil.

This study was conducted in the municipalities of southern Brazil, which included the states of Paraná (PR) with 399 municipalities distributed in 10 mesoregions, Santa Catarina (SC) with 295 municipalities distributed in six mesoregions, and Rio Grande do Sul (RS) with 497 municipalities distributed in seven mesoregions [26]. The southern states of Brazil are located between 22°30′ and 33°45′ S and 57°59′ and 48°00′ W, bordering Uruguay to the south, Argentina and Paraguay to the west, Atlantic Ocean to the east, and the central-western and south-eastern regions of Brazil to the north [26,27,28,29,30] (Figure 1). Southern states of Brazil form the smallest region, occupying just over 7% of the country’s land. The altitude ranges from sea level on the coast of the Atlantic Ocean to 1818 m in the mountains. The region comprises lakes, hills and mountains, coastal and interior plains, river valleys, and plateaus with spurs. This region was originally covered by Atlantic Forest in most areas and grassland vegetation in others. The climate ranges from tropical to temperate [26].

PR has a land area of 199,307.985 km^2^, with an estimated population of 11,597,484 inhabitants [26]. The average temperature in the state is 18.5 °C, with two predominant climate conditions: a tropical climate prevails in the northern, western, and coastal areas with an average temperature of 22 °C, whereas a subtropical to temperate climate prevails in the central–southern areas, with average temperatures of 10–22 °C [28].

SC, the smallest state of southern Brazil, has a land area of 95,737.895 km^2^ and an estimated population of 7,338,473 inhabitants [22]. The climate is humid subtropical with temperatures of 13–25 °C, and four well-defined seasons are recorded annually [27].

RS has an estimated population of 11,466,630 inhabitants and a land area of 281,707.156 km^2^ [26]. The climate is humid subtropical and mostly records hot summers; however, mild summers are recorded in a small area located in the northeast region at higher altitudes. There are average annual temperatures of 14–22 °C, and an average annual total precipitation from 1000 to >2000 mm [29].

### 2.2. Data acquisition

#### 2.2.1. Entomological Data on the *Ae. aegypti* Vector

Entomological data on *Ae. aegypti* were gathered using the Electronic System of the Citizen Information Service (Sistema Eletrônico do Serviço de Informações ao Cidadão; e-SIC), which is an MS secondary data source. Each state municipality was used as the unit of analysis to assess the vector presence. LIRAa data on the *Ae. aegypti* HI from 2003 to 2021 were collected. Given the lack of standardization among the municipalities regarding the period of *Ae. aegypti* HI surveys and the number of times that these surveys were performed per year, the last annual HI survey of each municipality was used to analyze the data (Supplementary Table S1). Using the HI, the percentage of properties recording positives for the presence of *Ae. aegypti* larvae can be calculated. Despite disregarding the number of positive containers or the vector production potential of each container, the HI is useful as it provides the percentage of positive properties in a region [4].

$$\mathrm{HI}=\frac{\mathrm{Positive}\;\mathrm{properties}}{\mathrm{Researched}\;\mathrm{properties}}\times 100$$

The data were organized cumulatively, with every HI value = 0 (zero) corresponding to the absence of the vector and HI value > 0 corresponding to the presence of the vector. The data for the municipalities that did not perform LIRAa were identified as “not reported.”

#### 2.2.2. Climatic Data

Correlations between the HI of *Ae. aegypti* with climatic and environmental variables were assessed from 2017 to 2021. This period was selected to include the highest number of municipalities that conducted the HI survey since, pursuant to Resolution No. 12 of 26 January 2017 [31], all municipalities are required to conduct annual surveys and monitor the *Ae. aegypti* vector [31]. The meteorological data, including the average maximum, minimum, and annual temperatures (°C), average relative humidity (%), and total precipitation (mm), were retrieved from the GIOVANNI web-based application (version 4.35, National Aeronautics and Space Administration) [32]. The mesoregions of each state (Supplementary Table S2) were assessed using time/seasonal series data. All months from 2017 to 2020 were selected to calculate average annual temperature and humidity and total annual precipitation values. The average annual, maximum, and minimum temperature data were expressed as “Surface air temperature” (MERRA-2 Model M2TMNXFLX v5.12.4), “2-m air temperature-daily max” (MERRA-2 Model M2TMNXFLX v5.12.4), and “2-m air temperature-daily min” (MERRA-2 Model M2TMNXFLX v5.12.4), respectively. The relative humidity data were expressed as “relative humidity” (AIRS AIRS3STM v7.0) and precipitation as “merged satellite-gauge precipitation estimate—final run mm/month” (GPM_3IMERGM v06). Supplementary Table S3 outlines the climatic data analyzed here.

#### 2.2.3. Environmental Data

Environmental data on land use and cover were retrieved from the Brazilian Annual Land Use and Cover Mapping Project (Projeto de Mapeamento Anual do Uso e Cobertura da Terra no Brasil; Mapbiomas) [33]. This monitoring is performed annually and the data are grouped by the mesoregion of each state. These land use and cover data are presented in five different classes (forest, non-forest natural formation, crop farming, livestock farming, non-vegetated area, and water bodies) and divided into subcategories based on the mosaic level (natural, anthropic, or unidentified) and the type of data (land use or cover). The following variables were used: agriculture, urban infrastructure, planted forest, and natural forest. Supplementary Table S3 outlines the environmental data that were sampled and analyzed here.

### 2.3. Statistical Analysis

The HI data were grouped by the average annual calculation of each mesoregion of three states in southern Brazil, to assess spatiotemporal patterns by year, state, and mesoregion. Significant differences between years (fixed factor: from 2017 to 2021), states (fixed factor: PR, SC, and RS), and mesoregions (random factor nested in states: 10 in PR, 6 in SC and 7 in RS) were identified by Permutational Multivariate Analysis of Variance (PERMANOVA), applied to Euclidian distance matrices with 9999 permutations of residuals under a reduced model [34]. Differences were considered significant when *p* < 0.05.

To evaluate relationships between the *Ae. aegypti* vector HI and climatic and environmental variables, multiple linear regression analysis was performed using the software Jamovi [35] with HI as the dependent variable, and average, maximum, and minimum temperatures, precipitation, humidity, natural forest, planted forest, agriculture, and urban infrastructure as independent variables. Relationships between variables were considered significant when *p* < 0.05. The values were previously log transformed (x + 1) to reduce data variability. To generate multiple regression models with different scales here, multicollinearity between independent variables was initially assessed using the regression test, considering variables with a relationship above 95%. No multicollinearity was found between variables (Supplementary Table S4). These relationships were considered significant when *p* < 0.05 in the analysis of variance (ANOVA). Due to the insufficient sample size, the state mesoregions were not analyzed.

Thematic maps of the spatiotemporal distribution of the *Ae. aegypti* vectors were drawn using HI data from 2003 to 2021, considering any value other than 0 corresponding to vector presence. The maps were cumulatively drawn to analyze the geographic expansion of the vector. These data were processed using Geographic Information System (Sistema de Informação Geográfica; SIG), QGIS software [36]. All shapefiles used here were derived from the Brazilian Institute of Geography and Statistics (Instituto Brasileiro de Geografia e Estatística; IBGE) and Datum Sirgas 2000 [37].

## 3. Results

### 3.1. Ae. aegypti Entomological Survey and Infestation

**Figure 2:**
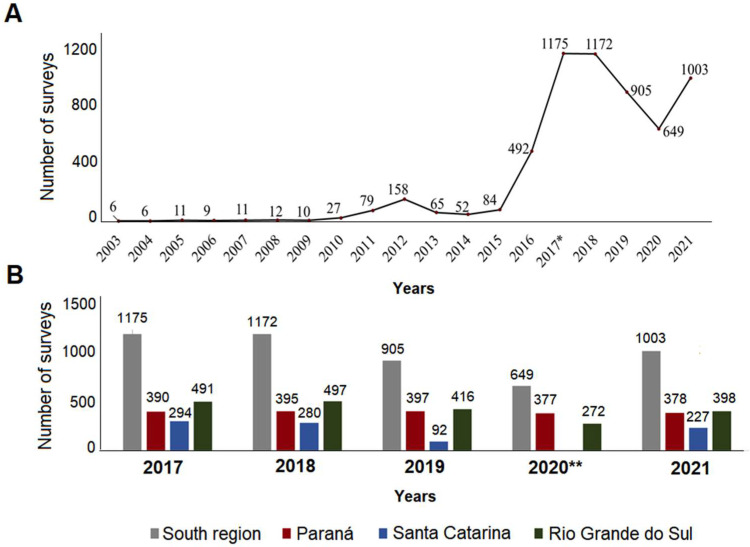
Number of house indices (HI) municipal surveys conducted between (**A**) 2003 and 2021 and (**B**) since 2017, pursuant to Resolution No. 12 of 26 January 2017 (Brasil, 2017). * Starting from that year, all municipalities are required to conduct annual surveys and monitor the Ae. aegypti vector (Resolution No. 12 of 26 January 2017). ** The state of Santa Catarina did not report *Ae. aegypti* HI data in 2020.

**Figure 3:**
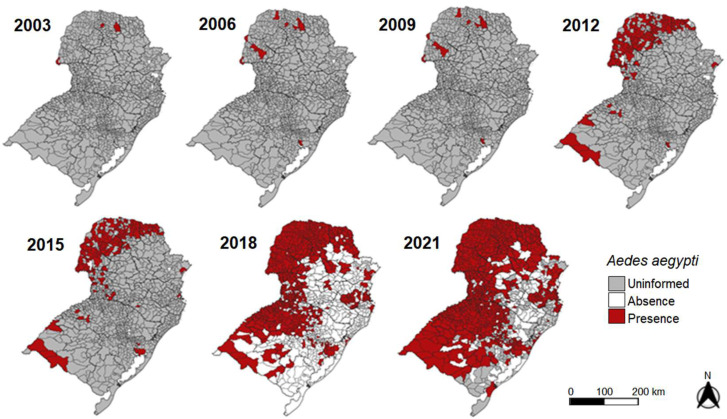
Confirmed presence, absence, and uninformed data of the *Ae. aegypti* vector based on the house indices (HI) surveys from 2003 to 2021. Confirmation data are presented cumulatively on spatiotemporal maps.

From 2003 to 2021, 5928 HI surveys were conducted in southern Brazil (Supplementary Table S1). The years with the highest numbers of HI surveys were 2017 and 2018, with 98.66% and 98.40% coverage, respectively (Figure 2). Conversely, the lowest numbers of HI surveys conducted were only 0.5% in 2003 and 2004 (Figure 2A). Since the requirement in 2017 that all municipalities conduct entomological surveys of the *Ae. aegypti* vector, 4906 surveys have been carried out with 2020 having the lowest (649) and 2017 having the highest (1175) number of surveys (Figure 2B). From 2003 to 2021, 829 (69.6%) municipalities confirmed the presence of the vector based on the HI survey (Figure 3). Supplementary Figure S1 shows all maps from 2003 to 2021.

In PR, 2671 surveys were conducted, accounting for 45.1% of the 5926 surveys conducted. In 2019, all municipalities in PR (399) reported HI results. The years with the lowest numbers of surveys were 2003 and 2004 (Supplementary Table S1). Throughout the study period, 350 (87.7%) of the municipalities confirmed the vector presence, accounting for 42.2% of the 829 confirmations in southern Brazil, (Figure 3). The mesoregions of the state with the highest number of confirmations were Norte Central Paranaense [North Central PR] (79), Noroeste Paranaense [Northwest PR] (61), and Oeste Paranaense [West PR] (50).

In SC, 934 surveys were conducted, accounting for 15.8% of the total conducted in the region. The year with the highest number of municipalities that conducted HI surveys was 2017 (294), with 99.66% of all municipalities of SC (Supplementary Figure S1 and Supplementary Table S1) conducting the surveys. Only the municipality of Florianopolis, which belongs to the Greater Florianopolis metropolitan area, did not report HI data in 2017. From 2005 to 2007, and in 2020, none of the SC municipalities reported HI data (Supplementary Figure S1). Of the 295 municipalities of SC, 143 confirmed the presence of the *Ae. aegypti* vector from 2003 to 2021, accounting for 17.23% of all confirmations the southern region and for 48.5% of the state’s municipalities. Of the 143 municipalities of SC, 80 (55%) belonged to the Oeste Catarinense mesoregion and 46 (32%) belonged to the Vale do Itajaí mesoregion (Figure 3).

In RS, 2323 (39.2%) HI surveys were recorded. In 2018, all 497 municipalities conducted this survey (Figure 3); however, in 2003, 2004, and 2009, no surveys were conducted (Figure 3 and Supplementary Figure S1). Of the 497 municipalities, 336 confirmed the presence of the *Ae. aegypti* vector, accounting for 67.6% of the municipalities of the state and for 40.5% of the 829 confirmations in southern Brazil (Figure 3, Supplementary Figure S1, and Supplementary Table S1). In 2005, the first HI survey was performed by the municipality of Porto Alegre, which remained the only municipality to conduct HI surveys on a regular basis until 2012. In this year, seven other municipalities also conducted HI surveys, namely Quaraí, Sant’Ana do Livramento, São Borja, and Uruguaiana, which belong to the Sudoeste Rio-Grandense [Southwest RS] mesoregion, and Ijuí, Santa Rosa and Santo Ângelo, which belong to the Noroeste Rio-grandense [Northwest RS] mesoregion.

**Figure 4:**
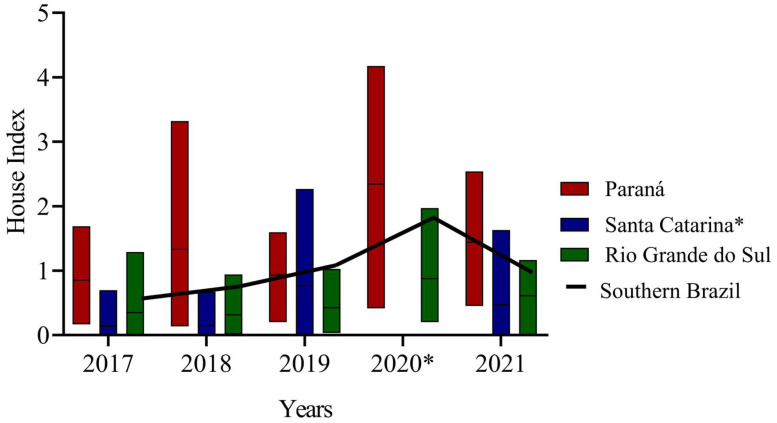
*Ae. aegypti* house indices (HI) survey results in Paraná, Santa Catarina and Rio Grande do Sul, from 2017 to 2021. The black line showed the mean of the entire southern region. States are represented by a minimum and maximum value with the central line indicating the average. * The state of Santa Catarina did not report HI data in 2020.

Although 2020 saw the lowest number of municipalities conducting these surveys in southern Brazil since 2017, the highest HI among sampled municipalities was also recorded in 2020 (HI = 2.28) (Figure 4). When considering the average HI per state, 2020 was also the year with the highest values recorded in PR (2.8) and RS (1.48). Conversely, 2019 was the year with the highest HI of 2.0 in SC (Figure 4).

The average annual *Ae. aegypti* HI was high in PR (averaging 1.67), moderate in SC (averaging 0.87), and low in RS (averaging 0.82). However, the PERMANOVA results showed that the HI variation between states depended on the year of analysis (significant interaction between state and year, Table 1). The HI was higher in PR than in SC only in 2017, 2018, and 2021 (Table 1 and Table 2; Figure 4). PR also recorded higher values than RS in all study years (Table 1 and Table 2; Figure 4). Conversely, the average annual *Ae. aegypti* HI did not significantly differ between SC and RS throughout the study period (Supplementary Tables S5 and S6 and Figure 4).

HI significantly varied between the mesoregions of each of the southern states of Brazil (Table 1). In PR, the highest HI values were recorded in the Centro Ocidental [Central-West PR], Noroeste [Northwest PR], Oeste [West PR], and Norte Central [Central-North PR] mesoregions, all of which had values higher than two (Supplementary Table S5). Conversely, the Sudeste [Southeast PR], Centro Oriental [Central-East PR], Centro-Sul [Central-South PR], and the Região Metropolitana de Curitiba [Metropolitan Area of Curitiba] had the lowest values (averaging 0.44). For SC, the mesoregions that recorded the highest mean HI values were Oeste Catarinense [West SC] (1.31) and Vale do Itajaí (0.61), and those with the lowest mean HI values were Sul Catarinense [South SC] and Mesorregião Serrana [Mountain Mesoregion] (averaging 0.015). In RS, the Noroeste Rio-grandense [Northwest RS] and Sudoeste Rio-grandense [Southwest RS] mesoregions had the highest mean HI values of 1.25 and 0.78, respectively. Conversely, the Nordeste Rio-grandense [Northeast RS] and Sudeste Rio-grandense [Southeast RS] mesoregions had the lowest mean values of 0.20 and 0.04, respectively (Supplementary Table S5).

### 3.2. Relationship with Climate and Environmental Factors

When evaluating the relationship between the infestation index, climate, and environmental variables, considering the collective data of the three states, we found significant differences in the model (*p* < 0,001) (Table 3). Individually for each state, significant differences were also observed in the three states, with climate and environmental variables explaining 76%, 77%, and 87% of the HI variability in PR, SC, and RS, respectively (Table 3).

Among climate variables showing significant associations in the model, precipitation was negatively correlated with the HI of southern Brazil (*p* = 0.030) and with that of PR (*p* = 0.027) (Table 3). The maximum temperature was positively correlated with the HI of RS (*p* = 0.002), whereas the minimum temperature was also positively correlated with the HI of the southern region (*p* = 0.032). Relative humidity was negatively correlated with the HI of the southern region (*p* < 0.001). Among the environmental variables with significant associations in the model, natural forest areas were negatively correlated with the infestation index of southern Brazil, whereas agricultural areas and urban infrastructure were positively correlated with the index. Agricultural areas were also positively correlated with the HI of RS, whereas areas with urban infrastructure were positively correlated with the HI of southern Brazil, PR, and RS (Table 3). Supplementary Table S6 presents all results from the model.

## 4. Discussion

There has been an increase in the number of municipalities infested by *Ae. aegypti* in the last five years (2017–2021) in southern Brazil, together with an increase in the average annual HI of these municipalities. Concomitantly, the number of confirmed cases of dengue has also been increasing in this region, which reported the third highest incidence in the country in 2021 (220.6 cases/100,000 inhabitants) [24]. According to Lee et al. [8], who evaluated the expansion of dengue in Brazil in the last 21 years, dengue outbreaks tend to increase once the vector is introduced in a given region, and the transmission zone is associated with temperature suitability, connectivity within the urban network, and urbanization [8]. Southern Brazil is known for its climatic barriers, which render the transmission of DENV and other arboviruses difficult because the seasonal temperatures are too low. These cold temperatures interfere with the *Ae. aegypti* life cycle and, consequently, hinder viral transmission [38]. However, the results from this study and the most recent epidemiological studies have recorded an increase in the number of dengue cases in the region, demonstrating that transmission is overcoming these barriers. This raises concern regarding control, monitoring, and surveillance measures [24]. Lins et al. [39] analyzed temperature extremes in southern Brazil between 1980 and 2016 and found an increase in the intensity of hot extreme temperatures in most of the region.

The results from this study also showed a considerable increase in the number of municipalities that have been conducting HI surveys since Resolution No. 12 of January 2017 came into effect [31]. Consequently, the number of municipalities that confirmed infestation with *Ae. aegypti* based on this entomological index increased, highlighting the spatiotemporal expansion of the vector, as shown here. Yet, despite this requirement, many municipalities failed to report HI data in LIRAa, possibly due to a lack of investment in entomological and epidemiological surveillance departments, leading to difficulties in staff retention and specialization and a shortage of adequate work equipment. Alternatively, the municipalities might have conducted other types of entomological surveys using larval traps or ovitraps. Therefore, entomological surveys conducted by Brazilian municipalities must be standardized for facilitating direct comparisons between their results.

The spatiotemporal expansion of the *Ae. aegypti* vector infestation in the municipalities analyzed here was heterogeneous. The mesoregions of PR, for example, had the highest HI averages and the highest numbers of municipalities that confirmed the presence of *Ae. aegypti*. The north of PR, the northernmost state of southern Brazil, is crossed by the Tropic of Capricorn, which determines the transition between tropical and subtropical climates. As a result of this geographic feature, different climate conditions are recorded within this state, with higher temperatures in the north and a typically subtropical climate in the remaining areas of the state [40]. This factor may affect the prevalence of *Ae. aegypti* in these areas. The distribution and abundance of this vector also depends on other elements that act together, including environmental and social factors [41,42,43,44,45,46]. Different climatic variables associated with an infestation in each state analyzed in this study were identified. Hence, even though the three states are located in the southern region, each state has its own environmental and climatic specificities, which can affect vector distribution dynamics.

Among the climatic variables that were correlated with the vector, precipitation was negatively correlated with the HI of the southern region and PR mesoregions, average maximum temperature was positively correlated with the HI of RS, average minimum temperature was positively correlated with the HI of the southern region, and the average relative humidity was negatively correlated with the HI of the south region. The effects of the same climatic variable can vary from region to region because the intensity of this variable can directly affect vector dynamics. For instance, rainfall can increase the mosquito density by providing more breeding sites for the vectors during their immature stages (e.g., containers with standing water). However, heavy rainfall events can reduce the density of mosquitoes by causing the same containers to overflow, rendering the growth of the larvae unfeasible [47]. Drought events can increase the abundance of mosquitoes, due to domestic water storage [48]. Ibarra et al. evaluated the dynamics of the *Ae. aegypti* vector distribution in an Ecuadorian city and identified that, in addition to climatic and seasonal effects, the vector populations are directly associated with social risk factors, such as access to piped water [46]. Temperature is another key external factor that interferes with mosquito biology and, consequently, with its distribution and population dynamics [45,49,50]. Temperature increases accelerate development cycles, thereby increasing populations [51]. The minimum temperature for *Ae. aegypti* development is 16 °C, and the maximum is 34 °C. However, other factors, in combination with temperature, can affect the larval development rate, as shown by Couret et al. (2014), who observed that nutrient availability and larval density also directly affect mosquito development [52].

Among the environmental variables, natural forest was negatively correlated with the HI of the southern region, crop farming was positively correlated with that of RS, and urban infrastructure was positively correlated with that of the southern region and of PR and RS. These results confirm the anthropophilic behavior of the *Ae. aegypti* species, which is extremely well adapted to the urban environment [42,46,53]. Changes attributed to urbanization have caused problems ranging from floods and water, soil, and air pollution to the formation of heat islands, the loss of native vegetation in and around cities, and the disappearance of important habitats for several species, including insect vectors, which may even intensify the proliferation of vectors and pathogens that cause infectious diseases [7,8,12,54].

There are some limitations to this study in that only a small sample size of the HI data was examined, especially in the years prior to 2017, which precluded correlation analyses with data from 2003 to 2016. In addition, the inconsistencies in municipal HI surveys over the years also prevented an analysis based on each municipality. SC, for example, did not report HI data in any municipality in 2020. A possible explanation for this is the COVID-19 pandemic, which also affected the entomological surveys of other municipalities in the southern region. In addition to social isolation, health measures against this new disease were intensified, and state epidemiological surveillance teams were mobilized to handle pandemic-related emergency situations [23]. In addition, other interesting variables for analysis, such as social variables, remain outdated because such information was collected in the last census conducted by IBGE in 2010. The present study is one of the first to historically analyze *Ae. aegypti* HI surveys in municipalities of southern Brazil, as well as the distribution of this vector and its correlation with climate and environmental variables. The results here demonstrate the increase in the distribution and infestation of the vector *Ae. aegypti,* in regions that were considered to have geographic barriers that hindered their presence and, consequently, the transmission of pathogens. In this way, epidemiological and entomological surveillance programs can be assisted in controlling the vector, *Ae. aegypti*, directing attention to these areas so that preventive measures can be taken.

## 5. Conclusions

Recently, the incidence of dengue has been increasing in southern Brazil, with a marked increase recorded in the number of municipalities infested with the *Ae. aegypti* vector. From 2003 to 2021, the number of *Ae. aegypti* HI surveys increased by 166.16% (from 6 to 1003). The number of municipalities deemed infested with the vector increased by 219.33% (from 3 to 661 municipalities), with the highest increase recorded in PR. Our model highlighted significant differences in the four groups analyzed (the southern region and its three states). Some environmental variables, such as urban infrastructure in the southern region and in PR and RS, were positively correlated with *Ae. aegypti* HI, highlighting the anthropophilic and urban character of the vector. Some climate variables were correlated with vector infestation, namely precipitation (positive correlation) and maximum and minimum temperatures (positive correlation) in RS and in the southern region, respectively, and humidity (negative correlation) in the southern region. The correlation of climate variables with vector distribution dynamics is complex because this correlation can be influenced by other factors, such as housing conditions, population density of immature *Ae. aegypti,* feeding in breeding sites, and rainfall intensity in some regions. Research assessing the spatiotemporal distribution of vectors, as undertaken by this study, is an important tool for developing planning and disease control strategies for public health.

## Figures and Tables

**Table 1:** PERMANOVA with the house indices (HI) of the southern region corresponding to the year (fixed factor: from 2017 to 2021), state (fixed factor: Paraná, Santa Catarina, and Rio Grande do Sul), and mesoregion (random factor nested in states: 10 in Paraná, 6 in Santa Catarina and 7 in Rio Grande do Sul). Df = degree of freedom, SS = sum of squares, MS = mean square, Pseudo-F = F statistic and p(perm) = PERMANOVA *p*-value.

Source	df	SS	MS	Pseudo-F	*p* (perm)
State	2	3.5726	1.7863	4.7848	0.019
Year	4	2.1326	0.53314	7.8968	0.001
Mesoregion (State)	20	7.8791	0.39395	5.8352	0.001
State X Year	7	1.1697	0.1671	2.4751	0.025
Res	107	19.382			

**Table 2:** Pairwise comparison of *Aedes aegypti* house indices (HI) between fixed groups (states and years) with significant differences [p(perms) ≤ 0.05].

Groups (Term State)	t	*p (perm)*
Paraná, Santa Catarina	2.8503	0.015
Paraná, Rio Grande do Sul	2.4083	0.03
**Groups (Terms Year)**	**t**	***p*** ***(perm)***
2017, 2019	3.0173	0.004
2017, 2020	5.4357	0.001
2017, 2021	6.0309	0.001
2018, 2020	6.2661	0.001
2018, 2021	3.3379	0.005
2019, 2020	4.8183	0.001
2020, 2021	3.8327	0.004
**Term ‘State × Year’ for pairs of levels of factor ‘State’**		
**Groups**	**t**	***p*** ***(perm)***
2017		
Paraná, Santa Catarina	5.0024	0.002
Paraná, Rio Grande do Sul	2.2776	0.031
2018		
Paraná, Santa Catarina	3.5714	0.007
Paraná, Rio Grande do Sul	2.7738	0.017
2020		
Paraná, Rio Grande do Sul	2.463	0.031
2021		
Paraná, Rio Grande do Sul	2.1969	0.013
Paraná, Santa Catarina	2.8811	0.009
**Term ‘State × Year’ for pairs of levels of factor ‘Year’**		
**Groups**	**t**	***p (perm)***
*Paraná*		
2017, 2018	2.2718	0.05
2017, 2020	7.6971	0.001
2017, 2021	7.8195	0.001
2018, 2020	6.8357	0.001
2019, 2020	3.7084	0.009
2020, 2021	4.9221	0.002
*Santa Catarina*		
2017, 2021	3.2204	0.026
2018, 2021	7.9261	0.004
*Rio Grande do Sul*		
2017,2019	2.5486	0.033
2017, 2020	4.4384	0.002
2017,2021	2.5116	0.038
2018,2019	3.2216	0.023
2018, 2020	5.0224	0.002
2019, 2020	4.0522	0.007

**Table 3:** Linear correlation analysis of the *Ae. aegypti* house indices (HI) with climate and environmental variables in southern Brazil The table presents only variables with significant differences. R = Pearson’s correlation coefficient, R^2^ = R-squared, F = ratio of two variances; df1 = Fisher’s F distribution 1, df2 = Fisher’s F distribution 2, *p* = *p*-value.

*Aedes aegypti*						
Region/State	R	R²	F	df1	df2	*p*
Southern region	0.85	0.72	27.46	9	98	<0.001
Paraná	0.87	0.76	13.82	9	40	< 0.001
Santa Catarina *	0.88	0.77	4.74	9	13	0.006
Rio Grande do Sul	0.93	0.87	18.49	9	25	< 0.001
**Significant Predictive Variables**						
**Predictor**	**Region/State**	**Estimate**	**SE**	**t**	***p***	**Stand.** **Estimate**
Annual precipitation	Southern region	−1.53	0.70	−2.21	0.030	−0.16
	Paraná	−3.77	1.65	−2.29	0.027	−0.37
Average maximum temperature	Rio Grande do Sul	14.13	4.09	3.45	0.002	0.66
Average minimum temperature	Southern region	4.18	1.93	2.17	0.032	0.20
Average humidity	Southern region	−9.57	1.80	−5.32	<0.001	−0.66
Natural forest	Southern region	−1.31	0.48	−2.71	0.008	−0.33
Agricultural	Rio Grande do Sul	1.11	0.50	2.19	0.038	0.64
Urban infrastructure	Southern region	1.12	0.28	4.00	<0.001	0.36
	Paraná	1.26	0.43	2.93	0.006	0.37
	Rio Grande do Sul	1.22	0.50	2.19	0.038	0.64

* The state of Santa Catarina did not report *Aedes aegypti* HI data in 2020.

## Data Availability

All relevant data are within the manuscript and its Supporting Information files.

## Supplementary Materials

The following supporting information can be downloaded at: https://www.mdpi.com/article/10.3390/tropicalmed8020077/s1, Figure S1: Municipalities of southern Brazil that confirmed the presence of the *Ae. aegypti* vector based on the HI survey from 2003 to 2021; Table S1: IIP of the municipalities in the southern region of Brazil from 2003 to 2021 used for the analyzes; Table S2: Coordinates of the mesoregions of southern Brazil, used for the acquisition of climatic data on the Giovanni platform; Table S3: Climatic and environmental variables used; Table S4: multicollinearity analyzes with climatic and environmental variables; Table S5: Average IIP values in the mesoregions of the states of the southern region of Brazil from 2017 to 2021; Table S6: PERMANOVA and Pair-Wise tests; Table S7: Linear models; Gif: Municipalities of southern Brazil, that confirmed the presence of the *Ae. aegypti* vector based on the HI survey from 2003 to 2021.
