# Supplementary material for: Aedes aegypti in Southern Brazil: Spatiotemporal Distribution Dynamics and Association with Climate and Environmental Factors
Source: Trop Med Infect Dis. 2023 Jan 20;8(2):77. doi: 10.3390/tropicalmed8020077 (PMC9961474; doi:10.3390/tropicalmed8020077)
Supplement: Supplementary file 1 [file tropicalmed-08-00077-s001.zip › Supplementary Table S2_ coordinates of the mesoregions.pdf]

Aedes aegypti **in the South Region of Brazil: Spatiotemporal Distribution Dynamics and Association with Climate and Environmental Factors**

Supplementary table 2 - Coordinates of the mesoregions of southern Brazil, used for the acquisition of climatic data on the Giovanni platform.

State	Mesoregion	Coordinates
**Paraná**	Noroeste Paranaense	"-54.1406,-24.2358,-52.0093,-23.5547"
	Centro Ocidental Paranaense	"-53.1519,-24.763,-52.009,-23.818"
	Norte Central Paranaense	"-52.1411,-24.873,-50.999,-23.379"
	Norte Pioneiro Paranaense	"-50.9106,-23.95,-49.724,-23.137"
	Centro Oriental Paranaense	"-51.2347,-25.2356,-49.411,-24.115"
	Oeste Paranaense	"-54.6185,-25.6311,-52.9486,-23.9832"
	Sudoeste Paranaense	"-53.9374,-26.3123,-52.4213,-25.5212"
	Centro-Sul Paranaense	"-53.1464,-25.9827,-51.2347,-24.6204"
	Sudeste Paranaense	"-51.5314,-26.3562,-50.0812,-24.8621"
	Metropolitana de Curitiba	"-50.213,-26.0266,-48.0157,-24.7522"
**Santa Catarina**	Oeste Catarinense	"-53.855,-27.2461,-50.7568,-26.2573"
	Norte Catarinense	"-51.2073,-26.6418,-48.5046,-25.9607"
	Serrana	"-51.5369,-28.2019,-49.6692,-26.8835"
	Vale do Itajaí	"-50.1526,-27.2791,-48.3948,-26.532"
	Grande Florianópolis	"-49.455,-27.9602,-48.3453,-27.2021"
	Sul Catarinense	"-49.9054,-29.1907,-48.587,-28.125"
**Rio Grande do Sul**	Noroeste Rio-grandense	“-55.0854, -28.872, -52.009, -27.554"
	Nordeste Rio-grandense	“-51.9434, -29.399, -50.098, -28.213”
	Centro Ocidental Rio-grandense	“-55.0745, -30.179, -53.361, -29.015”
	Centro Oriental Rio-grandense	“-52.9102, -30.52, -51.943, -29.356”
	Metropolitana de Porto Alegre	“-52.0752, -31.267, -50.208, -29.575”
	Sudoeste Rio-grandense	“-56.8652, -31.289, -53.789, -28.74”
	Sudeste Rio-grandense	“-53.7891, -33.618, -52.251, -30.718”

.
