# Supplementary material for: Aedes aegypti in Southern Brazil: Spatiotemporal Distribution Dynamics and Association with Climate and Environmental Factors
Source: Trop Med Infect Dis. 2023 Jan 20;8(2):77. doi: 10.3390/tropicalmed8020077 (PMC9961474; doi:10.3390/tropicalmed8020077)
Supplement: Supplementary file 1 [file tropicalmed-08-00077-s001.zip › Supplementary Table S4 _ multicollinearity analyzes with climatic and environmental variables_B.pdf]

Supplementary Table 4: multicollinearity analyzes with climatic and environmental variables

South region of Brazil. Correlations (Statistic collinearity) Marked correlations are significant at p < .05000 N=85 (Casewise deletion of missing data)

Variables	Annual precipitation	Annual average temperature	Average maximum temperature	Average minimum temperature	Average humidity (%)	Natural Forest	Planted forest	Agriculture	Urban infrastructure
Annual precipitation	1.0000<br>p= ---	-.3885<br>p=.000	-.5989<br>p=.000	-.1062<br>p=.274	.5089<br>p=.000	.3935<br>p=.000	.3696<br>p=.000	-.3995<br>p=.000	.1382<br>p=.154
Annual average temperature	-.3885<br>p=.000	1.0000<br>p= ---	.6619<br>p=.000	.4779<br>p=.000	-.5336<br>p=.000	-.5287<br>p=.000	-.5952<br>p=.000	.3778<br>p=.000	-.0624<br>p=.521
Average maximum temperature	-.5989<br>p=.000	.6619<br>p=.000	1.0000<br>p= ---	.3409<br>p=.000	-.7076<br>p=.000	-.4664<br>p=.000	-.5642<br>p=.000	.4421<br>p=.000	-.0712<br>p=.464
Average minimum temperature	-.1062<br>p=.274	.4779<br>p=.000	.3409<br>p=.000	1.0000<br>p= ---	.0982<br>p=.312	-.4188<br>p=.000	-.4853<br>p=.000	-.0735<br>p=.450	.3667<br>p=.000
Average humidity (%)	.5089<br>p=.000	-.5336<br>p=.000	-.7076<br>p=.000	.0982<br>p=.312	1.0000<br>p= ---	.3471<br>p=.000	.5055<br>p=.000	-.5866<br>p=.000	.4262<br>p=.000
Natural Forest	.3935<br>p=.000	-.5287<br>p=.000	-.4664<br>p=.000	-.4188<br>p=.000	.3471<br>p=.000	1.0000<br>p= ---	.7420<br>p=0.00	.0250<br>p=.797	.4134<br>p=.000
Planted forest	.3696<br>p=.000	-.5952<br>p=.000	-.5642<br>p=.000	-.4853<br>p=.000	.5055<br>p=.000	.7420<br>p=0.00	1.0000<br>p= ---	-.2383<br>p=.013	.1413<br>p=.145
Agriculture	-.3995<br>p=.000	.3778<br>p=.000	.4421<br>p=.000	-.0735<br>p=.450	-.5866<br>p=.000	.0250<br>p=.797	-.2383<br>p=.013	1.0000<br>p= ---	-.0666<br>p=.493
Urban infrastructure	.1382<br>p=.154	-.0624<br>p=.521	-.0712<br>p=.464	.3667<br>p=.000	.4262<br>p=.000	.4134<br>p=.000	.1413<br>p=.145	-.0666<br>p=.493	1.0000<br>p= ---

State of Paraná (PR). Correlations (PR STATISTIC collinearity) Marked correlations are significant at p < .05000 N=40 (Casewise deletion of missing data)

Variables	Annual precipitation	Annual average temperature	Average maximum temperature	Average minimum temperature	Average humidity (%)	Natural forest	Planted forest	Agriculture	Urban infrastructure
Annual precipitation	1.0000<br>p= ---	-.5552<br>p=.000	-.7072<br>p=.000	-.2652<br>p=.063	.7178<br>p=.000	.4895<br>p=.000	.4473<br>p=.001	-.2520<br>p=.077	.1129<br>p=.435
Annual average temperature	-.5552<br>p=.000	1.0000<br>p= ---	.6550<br>p=.000	.7639<br>p=.000	-.5580<br>p=.000	-.6744<br>p=.000	-.7800<br>p=.000	.5023<br>p=.000	.1618<br>p=.261
Average maximum temperature	-.7072<br>p=.000	.6550<br>p=.000	1.0000<br>p= ---	.4624<br>p=.001	-.5166<br>p=.000	-.4891<br>p=.000	-.5302<br>p=.000	.4619<br>p=.001	.1854<br>p=.198
Average minimum temperature	-.2652<br>p=.063	.7639<br>p=.000	.4624<br>p=.001	1.0000<br>p= ---	-.1800<br>p=.211	-.3893<br>p=.005	-.6600<br>p=.000	.4863<br>p=.000	.4210<br>p=.002
Average humidity (%)	.7178<br>p=.000	-.5580<br>p=.000	-.5166<br>p=.000	-.1800<br>p=.211	1.0000<br>p= ---	.6214<br>p=.000	.6529<br>p=.000	-.4212<br>p=.002	.3445<br>p=.014
Natural Forest	.4895<br>p=.000	-.6744<br>p=.000	-.4891<br>p=.000	-.3893<br>p=.005	.6214<br>p=.000	1.0000<br>p= ---	.8061<br>p=.000	-.3352<br>p=.017	.3369<br>p=.017
Planted forest	.4473<br>p=.001	-.7800<br>p=.000	-.5302<br>p=.000	-.6600<br>p=.000	.6529<br>p=.000	.8061<br>p=.000	1.0000<br>p= ---	-.4194<br>p=.002	.0208<br>p=.886
Agriculture	-.2520<br>p=.077	.5023<br>p=.000	.4619<br>p=.001	.4863<br>p=.000	-.4212<br>p=.002	-.3352<br>p=.017	-.4194<br>p=.002	1.0000<br>p= ---	.0211<br>p=.884
Urban infrastructure	.1129<br>p=.435	.1618<br>p=.261	.1854<br>p=.198	.4210<br>p=.002	.3445<br>p=.014	.3369<br>p=.017	.0208<br>p=.886	.0211<br>p=.884	1.0000<br>p= ---

State of Santa Catarina (SC). Correlations (SC STATISTIC collinearity) Marked correlations are significant at $p < .05000$ N=15 (Casewise deletion of missing data)

	Annual precipitation	Annual average temperature	Average maximum temperature	Average minimum temperature	Average humidity (%)	Natural forest	Planted forest	Agriculture	Urban infrastructure
Annual precipitation	1.0000	.0343	.0913	-.1205	.0538	.2100	.0661	-.3664	.1086
	p= ---	p=.877	p=.679	p=.584	p=.807	p=.336	p=.764	p=.086	p=.622
Annual average temperature	.0343	1.0000	.2753	.4808	.2004	-.1164	-.3086	.1174	.5572
	p=.877	p= ---	p=.204	p=.020	p=.359	p=.597	p=.152	p=.594	p=.006
Average maximum temperature	.0913	.2753	1.0000	-.0198	-.5052	.5420	.2833	.3649	.4926
	p=.679	p=.204	p= ---	p=.928	p=.014	p=.008	p=.190	p=.087	p=.017
Average minimum temperature	-.1205	.4808	-.0198	1.0000	.7472	-.7041	-.8862	-.2091	.7526
	p=.584	p=.020	p=.928	p= ---	p=.000	p=.000	p=.000	p=.338	p=.000
Average humidity (%)	.0538	.2004	-.5052	.7472	1.0000	-.7672	-.7940	-.5341	.3270
	p=.807	p=.359	p=.014	p=.000	p= ---	p=.000	p=.000	p=.009	p=.128
Natural forest	.2100	-.1164	.5420	-.7041	-.7672	1.0000	.9124	.5955	-.1683
	p=.336	p=.597	p=.008	p=.000	p=.000	p= ---	p=.000	p=.003	p=.443
Planted forest	.0661	-.3086	.2833	-.8862	-.7940	.9124	1.0000	.5627	-.5273
	p=.764	p=.152	p=.190	p=.000	p=.000	p=.000	p= ---	p=.005	p=.010
Agriculture	-.3664	.1174	.3649	-.2091	-.5341	.5955	.5627	1.0000	-.0337
	p=.086	p=.594	p=.087	p=.338	p=.009	p=.003	p=.005	p= ---	p=.879
Urban infrastructure	.1086	.5572	.4926	.7526	.3270	-.1683	-.5273	-.0337	1.0000
	p=.622	p=.006	p=.017	p=.000	p=.128	p=.443	p=.010	p=.879	p= ---

Correlations: State of Rio Grande do Sul (RS). Correlations (RS STATISTIC collinearity). Marked correlations are significant at p < .05000 N=28 (Casewise deletion of missing data)

	Annual precipitation	Annual average temperature	Average Maximum temperature	Average minimum temperature	Average humidity (%)	Natural forest	Planted forest	Agriculture	Urban infrastructure
Annual precipitation	1.0000<br>p= ---	-.0487<br>p=.781	-.3499<br>p=.039	.0821<br>p=.639	.1917<br>p=.270	.1469<br>p=.400	-.0145<br>p=.934	-.1525<br>p=.382	-.0632<br>p=.718
Annual average temperature	-.0487<br>p=.781	1.0000<br>p= ---	.5213<br>p=.001	.1730<br>p=.320	-.4358<br>p=.009	-.2954<br>p=.085	-.3383<br>p=.047	.4092<br>p=.015	-.1567<br>p=.369
Average maximum temperature	-.3499<br>p=.039	.5213<br>p=.001	1.0000<br>p= ---	-.1016<br>p=.561	-.8496<br>p=.000	-.2662<br>p=.122	-.7447<br>p=.000	.6108<br>p=.000	-.2139<br>p=.217
Average minimum temperature	.0821<br>p=.639	.1730<br>p=.320	-.1016<br>p=.561	1.0000<br>p= ---	.4295<br>p=.010	.1938<br>p=.265	.0932<br>p=.594	.0457<br>p=.794	.6281<br>p=.000
Average humidity (%)	.1917<br>p=.270	-.4358<br>p=.009	-.8496<br>p=.000	.4295<br>p=.010	1.0000<br>p= ---	.2387<br>p=.167	.7682<br>p=.000	-.6732<br>p=.000	.4279<br>p=.010
Natural Forest	.1469<br>p=.400	-.2954<br>p=.085	-.2662<br>p=.122	.1938<br>p=.265	.2387<br>p=.167	1.0000<br>p= ---	.3764<br>p=.026	.3193<br>p=.062	.7502<br>p=.000
Planted forest	-.0145<br>p=.934	-.3383<br>p=.047	-.7447<br>p=.000	.0932<br>p=.594	.7682<br>p=.000	.3764<br>p=.026	1.0000<br>p= ---	-.4681<br>p=.005	.4330<br>p=.009
Agriculture	-.1525<br>p=.382	.4092<br>p=.015	.6108<br>p=.000	.0457<br>p=.794	-.6732<br>p=.000	.3193<br>p=.062	-.4681<br>p=.005	1.0000<br>p= ---	.0675<br>p=.700
Urban infrastructure	-.0632<br>p=.718	-.1567<br>p=.369	-.2139<br>p=.217	.6281<br>p=.000	.4279<br>p=.010	.7502<br>p=.000	.4330<br>p=.009	.0675<br>p=.700	1.0000<br>p= ---
