# Supplementary material for: Aedes aegypti in Southern Brazil: Spatiotemporal Distribution Dynamics and Association with Climate and Environmental Factors
Source: Trop Med Infect Dis. 2023 Jan 20;8(2):77. doi: 10.3390/tropicalmed8020077 (PMC9961474; doi:10.3390/tropicalmed8020077)
Supplement: Supplementary file 1 [file tropicalmed-08-00077-s001.zip › Supplementary Table S5_ Average IIP values in the mesoregions of the states of the southern region of Brazil from 2017 to 2021_B.pdf]

Supplementary Table 5: Average IIP values in the mesoregions of the states of the southern region of Brazil from 2017 to 2021.

**State** (Mesoregion)	**2017**	**2018**	**2019**	**2020**	**2021**	**total**
**Paraná**	**1.033826**	**1.675599**	**1.09469**	**2.857137**	**1.693834**	**1.67102**
Norte Pioneiro Paranaense	0.835634	1.71092	0.9008985	2.614365	1.310721	1.474508
Centro Ocidental Paranaense	1.35386	1.896351	1.5958171	3.967674	2.237045	2.210149
Centro Oriental Paranaense	0.195	0.185714	0.2	1.230769	0.707143	0.503725
Centro-Sul Paranaense	0.2475	0.337866	0.2573916	0.665858	0.519592	0.405642
Metropolitana de Curitiba	0.333	0.136151	0.4929658	0.413961	0.452642	0.365744
Noroeste Paranaense	1.512775	3.320543	1.5142685	4.174536	2.482385	2.600901
Norte Central Paranaense	1.22688	2.016405	1.3963533	3.984121	1.957049	2.116162
Oeste Paranaense	1.688005	2.19117	1.4169144	3.46923	2.53871	2.260806
Sudeste Paranaense	0.166667	0.409524	0.3428571	1.070086	0.594499	0.516727
Sudoeste Paranaense	0.997289	1.105587	1.2164566	1.833754	1.615904	1.353798
**Santa Catarina**	**0.29932**	**0.319286**	**2.001087**	**-**	**0.882379**	**0.87552**
Grande Florianópolis	0	0.03125	0.1666667	-	0.436364	0.15857
Norte Catarinense	0.011538	0.003846	0.45	-	0.16087	0.156564
Oeste Catarinense	0.698305	0.674576	2.2662162	-	1.632039	1.317784
Serrana	0	0	0	-	0	0
Sul Catarinense	0	0	0.0666667	-	0.058621	0.031322
Vale do Itajaí	0.098148	0.184	1.6444444	-	0.55	0.619148
**Rio Grande do Sul**	**0.626489**	**0.498189**	**0.687081**	**1.488971**	**0.837688**	**0.82768**
Metropolitana de Porto Alegre	0.253608	0.184694	0.4724638	0.816667	0.589474	0.463381
Centro Ocidental Rio-grandense	0.348387	0.270968	0.5821429	0.942857	0.437931	0.516457
Centro Oriental Rio-grandense	0.061111	0.037037	0.1794872	0.7125	0.640476	0.326122
Nordeste Rio-grandense	0.045283	0.012963	0.0628571	0.557143	0.357576	0.207164
Noroeste Rio-grandense	1.229327	0.943519	1.0283019	1.970186	1.083412	1.250949
Sudeste Rio-grandense	0	0.012	0.0333333	0.2	0	0.049067
Sudoeste Rio-grandense	0.436842	0.752632	0.6176471	0.935294	1.164706	0.781424
