# Supplementary material for: Aedes aegypti in Southern Brazil: Spatiotemporal Distribution Dynamics and Association with Climate and Environmental Factors
Source: Trop Med Infect Dis. 2023 Jan 20;8(2):77. doi: 10.3390/tropicalmed8020077 (PMC9961474; doi:10.3390/tropicalmed8020077)
Supplement: Supplementary file 1 [file tropicalmed-08-00077-s001.zip › Supplementary Table S6_Permanova and Pair-Wise tests_.pdf]

Supplementary table 6: Permanova and Pair-Wise tests

PERMANOVA table of results

Source	df	SS	MS	Pseudo-F	*P(perm)*
State	2	35,726	17,863	47,848	0,019
Year	4	21,326	0,53314	78,968	0,001
Mesoregion (State)	20	78,791	0,39395	58,352	0,001
State x Year	7	11,697	0,1671	24,751	0,025
Residuos	dios74	4,996	0,067514		
Total	107	19,382			

PAIR-WISE TESTS

Term ' State x Year ' for pairs of levels of factor 'STATE'

Within level '2017' of factor 'YEAR'

Groups	t	*P(perm)*
Paraná, Rio Grande do Sul	22,776	0,031
Paraná, Santa Catarina	50,024	0,002
Rio Grande do Sul, Santa Catarina	20,805	0,077

Within level '2018' of factor 'Year'

Groups	t	*P(perm)*
Paraná, Rio Grande do Sul	27,738	0,017
Paraná, Santa Catarina	35,714	0,007
Rio Grande do Sul, Santa Catarina	11,483	0,273

Within level '2019' of factor 'Year'

Groups	t	*P(perm)*
Paraná, Rio Grande do Sul	18,844	0,066
Paraná, Santa Catarina	0,097504	0,939
Rio Grande do Sul, Santa Catarina	0,44803	0,78

Within level '2020' of factor 'Year'

Groups	t	*P(perm)*
Paraná, Rio Grande do Sul	2,463	0,031

Within level '2021' of factor 'Year'

Groups	t	*P(perm)*
Paraná, Rio Grande do Sul	21,969	0,013
Paraná, Santa Catarina	28,811	0,009
Rio Grande do Sul, Santa Catarina	0,57756	0,569

Term ' State x Year' for pairs of levels of factor 'YEAR'

Within level 'Paraná' of factor 'State'

**Groups**	**t**	***P(perm)***
2017, 2018	22,718	0,05
2017, 2019	17,692	0,106
2017, 2020	76,971	0,001
2017, 2021	78,195	0,001
2018, 2019	0,052789	0,962
2018, 2020	68,357	0,001
2018, 2021	18,577	0,094
2019, 2020	37,084	0,009
2019, 2021	14,653	0,184
2020, 2021	49,221	0,002

Within level 'Rio Grande do Sul' of factor 'State'

**Groups**	**t**	***P(perm)***
2017, 2018	0,20338	0,861
2017, 2019	25,486	0,033
2017, 2020	44,384	0,002
2017, 2021	25,116	0,038
2018, 2019	32,216	0,023
2018, 2020	50,224	0,002
2018, 2021	14,339	0,198
2019, 2020	40,522	0,007
2019, 2021	0,14152	0,913
2020, 2021	18,193	0,072

Within level 'Santa Catarina' of factor 'State'

**Groups**	**t**	***P(perm)***
2017, 2018	13,776	0,234
2017, 2019	15,963	0,132
2017, 2021	32,204	0,026
2018, 2019	14,895	0,187
2018, 2021	79,261	0,004
2019, 2021	0,75726	0,546

**Term 'Mesoregion(State)**		
Within level 'Paraná' of factor 'State'		
**Groups**	**t**	***P(perm)***
Centro Ocidental Paranaense, Centro Oriental Paranaense	50,667	0,004
Centro Ocidental Paranaense, Centro-Sul Paranaense	59,867	0,004
Centro Ocidental Paranaense, Metropolitana de Curitiba	78,603	0,002
Centro Ocidental Paranaense, Noroeste Paranaense	0,27293	0,775
Centro Ocidental Paranaense, Norte Central Paranaense	10,866	0,287
Centro Ocidental Paranaense, Norte Pioneiro Paranaense	22,948	0,082
Centro Ocidental Paranaense, Oeste Paranaense	0,68039	0,535
Centro Ocidental Paranaense, Sudeste Paranaense	58,414	0,007
Centro Ocidental Paranaense, Sudoeste Paranaense	3,114	0,028
Centro Oriental Paranaense, Centro-Sul Paranaense	0,15417	0,914
Centro Oriental Paranaense, Metropolitana de Curitiba	0,31395	0,792
Centro Oriental Paranaense, Noroeste Paranaense	7,472	0,001
Centro Oriental Paranaense, Norte Central Paranaense	90,364	0,003
Centro Oriental Paranaense, Norte Pioneiro Paranaense	52,247	0,01
Centro Oriental Paranaense, Oeste Paranaense	9,427	0,003
Centro Oriental Paranaense, Sudeste Paranaense	0,61345	0,576
Centro Oriental Paranaense, Sudoeste Paranaense	51,138	0,004
Centro-Sul Paranaense, Metropolitana de Curitiba	0,31108	0,763
Centro-Sul Paranaense, Noroeste Paranaense	12,641	0,002
Centro-Sul Paranaense, Norte Central Paranaense	13,443	0,002
Centro-Sul Paranaense, Norte Pioneiro Paranaense	9,622	0,002
Centro-Sul Paranaense, Oeste Paranaense	36,676	0,002
Centro-Sul Paranaense, Sudeste Paranaense	10,216	0,367
Centro-Sul Paranaense, Sudoeste Paranaense	12,925	0,001
Metropolitana de Curitiba, Noroeste Paranaense	55,576	0,009
Metropolitana de Curitiba, Norte Central Paranaense	57,913	0,012
Metropolitana de Curitiba, Norte Pioneiro Paranaense	41,606	0,024
Metropolitana de Curitiba, Oeste Paranaense	71,225	0,011
Metropolitana de Curitiba, Sudeste Paranaense	0,63864	0,563
Metropolitana de Curitiba, Sudoeste Paranaense	89,455	0,003
Noroeste Paranaense, Norte Central Paranaense	23,603	0,094
Noroeste Paranaense, Norte Pioneiro Paranaense	11,106	0,003
Noroeste Paranaense, Oeste Paranaense	11,786	0,303
Noroeste Paranaense, Sudeste Paranaense	13,324	0,002
Noroeste Paranaense, Sudoeste Paranaense	32,312	0,02
Norte Central Paranaense, Norte Pioneiro Paranaense	67,656	0,004
Norte Central Paranaense, Oeste Paranaense	11,358	0,326
Norte Central Paranaense, Sudeste Paranaense	18,12	0,001
Norte Central Paranaense, Sudoeste Paranaense	2,603	0,033
Norte Pioneiro Paranaense, Oeste Paranaense	51,298	0,011
Norte Pioneiro Paranaense, Sudeste Paranaense	10,384	0,001
Norte Pioneiro Paranaense, Sudoeste Paranaense	0,16882	0,904
Oeste Paranaense, Sudeste Paranaense	14,238	0,001

Oeste Paranaense, Sudoeste Paranaense	41,925	0,023
Sudeste Paranaense, Sudoeste Paranaense	66,159	0,001

Within level 'Rio Grande do Sul' of factor 'State'		
**Groups**	**t**	***P(perm)***
Centro Ocidental Rio-grandense, Centro Oriental Rio-grandense	21,846	0,077
Centro Ocidental Rio-grandense, Metropolitana de Porto Alegre	0,38561	0,722
Centro Ocidental Rio-grandense, Nordeste Rio-grandense	35,296	0,028
Centro Ocidental Rio-grandense, Noroeste Rio-grandense	78,199	0,002
Centro Ocidental Rio-grandense, Sudeste Rio-grandense	5,421	0,002
Centro Ocidental Rio-grandense, Sudoeste Rio-grandense	19,463	0,125
Centro Oriental Rio-grandense, Metropolitana de Porto Alegre	25,013	0,077
Centro Oriental Rio-grandense, Nordeste Rio-grandense	45,547	0,01
Centro Oriental Rio-grandense, Noroeste Rio-grandense	44,479	0,002
Centro Oriental Rio-grandense, Sudeste Rio-grandense	28,334	0,061
Centro Oriental Rio-grandense, Sudoeste Rio-grandense	33,931	0,016
Metropolitana de Porto Alegre, Nordeste Rio-grandense	3,73	0,037
Metropolitana de Porto Alegre, Noroeste Rio-grandense	44,802	0,005
Metropolitana de Porto Alegre, Sudeste Rio-grandense	47,624	0,005
Metropolitana de Porto Alegre, Sudoeste Rio-grandense	19,958	0,115
Nordeste Rio-grandense, Noroeste Rio-grandense	5,931	0,001
Nordeste Rio-grandense, Sudeste Rio-grandense	21,779	0,106
Nordeste Rio-grandense, Sudoeste Rio-grandense	44,063	0,003
Noroeste Rio-grandense, Sudeste Rio-grandense	67,581	0,001
Noroeste Rio-grandense, Sudoeste Rio-grandense	25,223	0,057
Sudeste Rio-grandense, Sudoeste Rio-grandense	50,587	0,003

Within level 'Santa Catarina' of factor 'State'		
**Groups**	**t**	***P(perm)***
Grande Florianópolis, Norte Catarinense	0,99122	0,473
Grande Florianópolis, Oeste Catarinense	0,034484	0,97
Grande Florianópolis, Serrana	14,151	0,241
Grande Florianópolis, Sul Catarinense	13,444	0,205
Grande Florianópolis, Vale do Itajaí	0,8962	0,535
Norte Catarinense, Oeste Catarinense	98,699	0,001
Norte Catarinense, Serrana	41,429	0,04
Norte Catarinense, Sul Catarinense	25,639	0,113
Norte Catarinense, Vale do Itajaí	0,076142	0,916
Oeste Catarinense, Serrana	11,828	0,029
Oeste Catarinense, Sul Catarinense	76,701	0,013
Oeste Catarinense, Vale do Itajaí	3,632	0,032
Serrana, Sul Catarinense	1	1
Serrana, Vale do Itajaí	19,467	0,16
Sul Catarinense, Vale do Itajaí	27,356	0,062
