# Supplementary material for: Aedes aegypti in Southern Brazil: Spatiotemporal Distribution Dynamics and Association with Climate and Environmental Factors
Source: Trop Med Infect Dis. 2023 Jan 20;8(2):77. doi: 10.3390/tropicalmed8020077 (PMC9961474; doi:10.3390/tropicalmed8020077)
Supplement: Supplementary file 1 [file tropicalmed-08-00077-s001.zip › Supplementary Table S7_ Linear Models.pdf]

Supplementary table 7: Linear models

Aedes aegypti						
**Region/State**	**R**	**R²**	**F**	**df1**	**df2**	***p***
South region	0.85	0.72	27.46	9	98	<.001
Paraná	0.87	0.76	13.82	9	40	< .001
Santa Catarina*	0.88	0.77	4.74	9	13	0.006
Rio Grande do Sul	0.93	0.87	18.49	9	25	< .001

Significant predictive variables

**Predictor**	**Region/State**	**Estimate**	**SE**	**t**	***p***	**Stand. Estimate**
Annual precipitation	South region	-1.53	0.70	-2.21	0.030	-0.16
	Paraná	-3.77	1.65	-2.29	0.027	-0.37
Average maximum temperature	Rio Grande do Sul	14.13	4.09	3.45	0.002	0.66
Average minimum temperature	South region	4.18	1.93	2.17	0.032	0.20
Average humidity	South region	-9.57	1.80	-5.32	<.001	-0.66
Natural Forest	South region	-1.31	0.48	-2.71	0.008	-0.33
Agriculture	Rio Grande do Sul	1.11	0.50	2.19	0.038	0.64
Urban infrastructure	South region	1.12	0.28	4.00	<.001	0.36
	Paraná	1.26	0.43	2.93	0.006	0.37
	Rio Grande do Sul	1.22	0.50	2.19	0.038	0.64

All predictive variables

**Predictor**	**Region/State**	**Estimate**	**SE**	**t**	***p***	**Stand. Estimate**
Annual precipitation	South Region	-1.53	0.70	-2.21	0.030	-0.16
	Paraná	-3.77	1.65	-2.29	0.027	-0.37
	Santa Catarina	-3.39	2.14	-1.59	0.136	-0.36
	Rio Grande do Sul	0.21	0.68	0.31	0.763	0.04
Average Temperature	South Region	-0.59	2.07	-2.08	0.778	-0.2
	Paraná	4.75	6.19	0.74	0.465	0.14
	Santa Catarina	9.35	5.18	1.80	0.094	0.31
	Rio Grande do Sul	-1.82	1.06	-1.72	0.097	-0.18
Average maximum temperature	South Region	1.89	3.23	0.58	0.560	0.06
	Paraná	-5.83	5.03	-1.16	0.253	-0.17
	Santa Catarina	39.13	19.54	2.00	0.067	0.87
	Rio Grande do Sul	14.13	4.09	3.45	0.002	0.66
Average minimum temperature	South region	4.18	1.93	2.17	0.032	0.20
	Paraná	-4.39	5.71	-0.77	0.446	-0.15
	Santa Catarina	10.77	22.09	0.49	0.634	0.85
	Rio Grande do Sul	-5.05	4.16	-1.21	0.236	-0.37

Average humidity	South region	-9.57	1.80	-5.32	<.00<br>1	-0.66
	Paraná	-5.25	4.87	-1.08	0.287	-0.21
	Santa Catarina	-3.88	8.52	-0.46	0.656	-0.32
	Rio Grande do Sul	0.31	2.53	0.12	0.902	0.05
Natural Forest	South region	-1.31	0.48	-2.71	0.008	-0.33
	Paraná	-0.65	0.71	-0.92	0.364	-0.17
	Santa Catarina	6.77	4.65	1.46	0.169	1.59
	Rio Grande do Sul	-1.57	1.02	-1.54	0.136	-0.53
Planted Forest	South region	0.18	0.19	0.92	0.361	0.10
	Paraná	-0.57	0.42	-1.37	0.179	-0.32
	Santa Catarina	-2.08	2.02	-1.03	0.323	-124
	Rio Grande do Sul	0.20	0.21	0.96	0.347	0.18
Agriculture	South region	-0.09	0.20	-0.43	0.671	-0.03
	Paraná	0.66	0.56	1.16	0.251	0.13
	Santa Catarina	-0.68	1.12	-0.61	0.551	-0.44
	Rio Grande do Sul	1.11	0.50	2.19	0.038	0.64
Urban infrastructure	South region	1.12	0.28	4.00	<.00<br>1	0.36
	Paraná	1.26	0.43	2.93	0.006	0.37
	Santa Catarina	-4.32	2.17	-1.99	0.068	-1.38
	Rio Grande do Sul	1.22	0.50	2.19	0.038	0.64

* The state of Santa Catarina did not report *Ae. aegypti* HI data in 2020
