# Supplementary figures and images for: Aedes aegypti in Southern Brazil: Spatiotemporal Distribution Dynamics and Association with Climate and Environmental Factors
Source: Trop Med Infect Dis. 2023 Jan 20;8(2):77. doi: 10.3390/tropicalmed8020077 (PMC9961474; doi:10.3390/tropicalmed8020077)

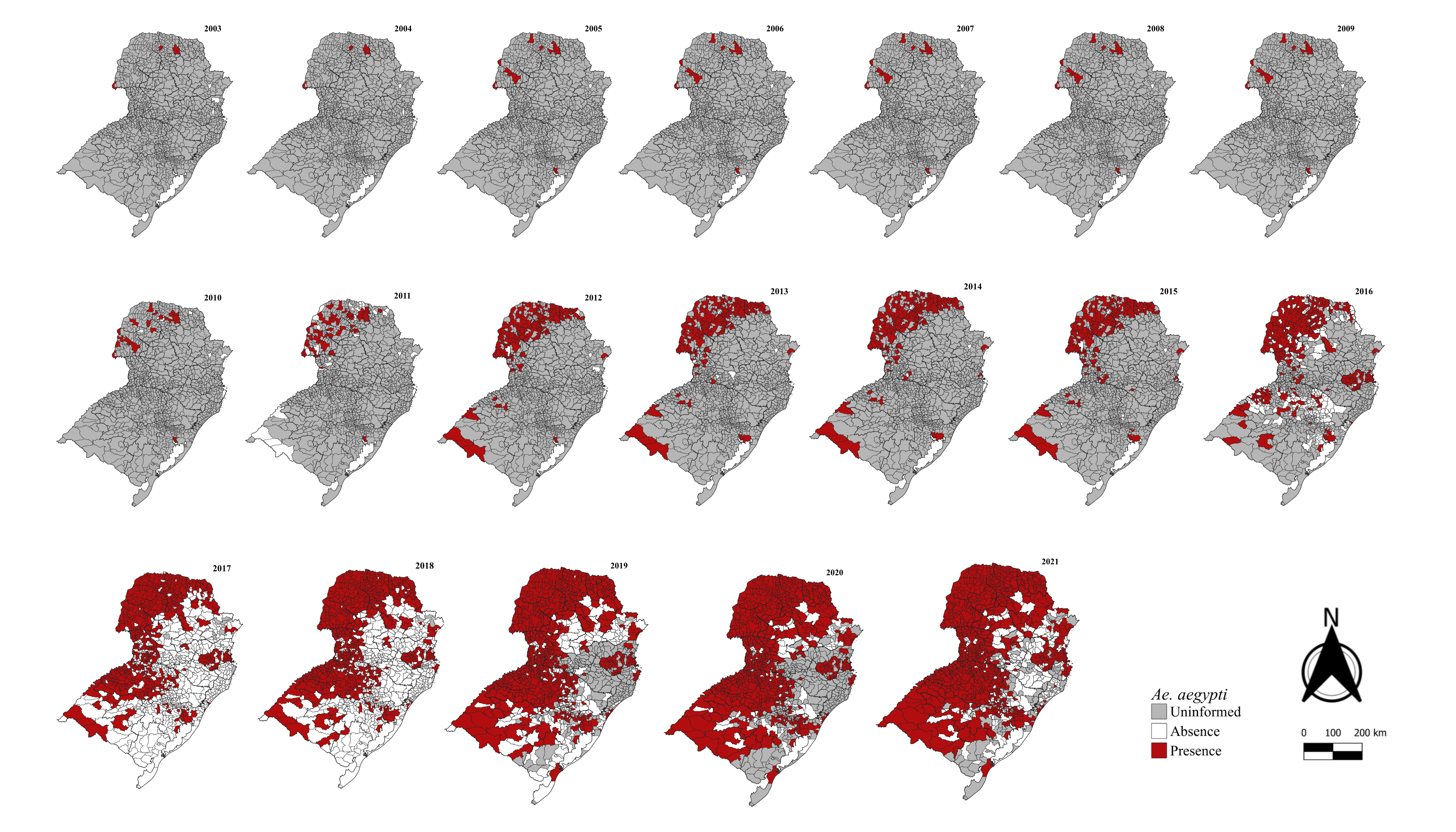

Supplement: Supplementary file 1 [file tropicalmed-08-00077-s001.zip › Supplementary Figure S1.png]
